# Evaluating complementary and alternative medicine interventions: in search of appropriate patient-centered outcome measures

**DOI:** 10.1186/1472-6882-6-38

**Published:** 2006-11-21

**Authors:** Marja J Verhoef, Laura C Vanderheyden, Trish Dryden, Devon Mallory, Mark A Ware

**Affiliations:** 1 Departments of Community Health Sciences and Medicine, Faculty of Medicine, University of Calgary, 3330/50 Hospital Drive NW, Calgary, AB, T2N 4N1, Canada; 2 Department of Community Health Sciences, Faculty of Medicine, University of Calgary, 33300 Hospital Drive NW, Calgary, AB, T2N 4N1, Canada; 3 Centre for Applied Research in Health, Technology & Education, School of Community and Health Studies, Centennial HP Science and Technology Centre, PO Box 631, Station A, Scarborough, ON, M1K 5E9, Canada; 4 Departments of Anesthesia and Family Medicine, McGill University, Room E19.145, Montreal General Hospital, 1650 Cedar Avenue, Montreal, QC, H3G 1A4, Canada

## Abstract

**Background:**

Central to the development of a sound evidence base for Complementary and Alternative Medicine (CAM) interventions is the need for valid, reliable and relevant outcome measures to assess whether the interventions work. We assessed the specific needs for a database that would cover a wide range of outcomes measures for CAM research and considered a framework for such a database.

**Methods:**

The study was a survey of CAM researchers, practitioners and students. An online questionnaire was emailed to the members of the Canadian Interdisciplinary Network for CAM Research (IN-CAM) and the CAM Education and Research Network of Alberta (CAMera). The majority of survey questions were open-ended and asked about outcome measures currently used, outcome measures' assessment criteria, sources of information, perceived barriers to finding outcome measures and outcome domains of importance. Descriptive quantitative analysis and qualitative content analysis were used.

**Results:**

One hundred and sixty-four completed surveys were received. Of these, 62 respondents reported using outcome measures in their CAM research and identified 92 different specific outcomes. The most important barriers were the fact that, for many health concepts, outcome measures do not yet exist, as well as issues related to accessibility of instruments. Important outcome domains identified included physical, psychological, social, spiritual, quality of life and holistic measures. Participants also mentioned the importance of individualized measures that assess unique patient-centered outcomes for each research participant, and measures to assess the context of healing and the process of healing.

**Conclusion:**

We have developed a preliminary framework that includes all components of health-related outcomes. The framework provides a foundation for a larger, comprehensive collection of CAM outcomes. It fits very well in a whole systems perspective, which requires an expanded set of outcome measures, such as individualized and holistic measures, with attention to issues of process and context.

## Background

Over the past 15 – 20 years, Complementary and Alternative Medicine (CAM) use has increased dramatically. Not surprisingly, questions of whether CAM interventions are safe, efficacious and effective have become key research issues. A review of articles indexed in MEDLINE between 1966 and 1996 suggests that both the number and proportion of reports of CAM clinical trials is increasing at a significant rate [1]. More recently, there is increasing attention to Integrative Medicine (IM), which combines mainstream medical therapies and CAM therapies for which there is some high-quality scientific evidence of safety and effectiveness [2].

Central to the development of a sound evidence base for CAM interventions is the need for valid, reliable and relevant outcome measures to assess whether the interventions work, in what conditions and for what population groups. The number of available patient-centered outcome measures from a variety of health and social science disciplines is enormous. Several books have been published on health outcome measures (e.g., [3-9]) and, more recently, several websites have been developed to provide brief overviews of outcome measures (e.g., Measurement Excellence and Training Resource Information Center (METRIC); Patient-Reported Outcome and Quality of Life Instruments Database (PROQOLID)). Despite these developments, there is growing recognition by CAM practitioners and researchers that the current array of outcome measures is not sufficient for use in CAM research and practice, as they do not cover the full spectrum of observed treatment effects.

Many CAM interventions (e.g., traditional Chinese medicine, chiropractic, naturopathy, massage therapy) may be conceptualized as whole systems of care [10-14] which consist of multiple components that provide an internally consistent, individualized approach to treatment. Components of care include, for example, the intervention itself as well as the process and context of healing, which are intertwined in a whole system based on a unique and consistent philosophical foundation. An acknowledgement of the whole systems' nature of CAM interventions (and of IM) requires an expanded set of outcome measures, of which survival, conventional assessments of biomedical outcomes and quality of life are only part. CAM interventions and IM are often aimed at affecting more than one aspect of a patient's life, and instead focus on maximizing the individual patient's capacity to achieve mental and physical balance and to, globally, restore his/her own health [11,13]. Thus, both individualized and global outcome measures appear necessary. It is becoming increasingly clear that commonly used outcome measures fall short in addressing these aspects. This perspective has led to a search for a broader range of outcome measures which may be less well-known, and for which psychometric quality is not always clear. To help raise awareness of the range of relevant and available outcome measures, we set out to develop an online database of outcome measures relevant to CAM/IM interventions which would be widely available. Here we present the results of a survey aimed at identifying the specific needs for a database that would cover a wide range of outcomes measures for CAM research. The objectives of the study were to identify: 1) where CAM researchers look for outcome measures; 2) which specific outcome measures are in use for CAM research; 3) assessment criteria used when selecting outcome measures; 4) perceived barriers to finding outcome measures for CAM research; and 5) outcome domains that are important for CAM research.

## Methods

The study design was a survey of CAM researchers, practitioners and students. The need for such a survey and the identification of relevant questions was pre-tested in a workshop on CAM outcome measures offered at the second Annual Symposium of the Canadian Interdisciplinary Network for Complementary and Alternative Medicine Research (IN-CAM) in November 2005. Thirty participants attended the workshop and as part of the workshop completed a brief paper and pencil questionnaire. The responses were used to refine the final questionnaire; for example, small changes were made to make the questionnaire more "practitioner-friendly" (some practitioner participants felt the questionnaire was not applicable to them as they did not conduct academically oriented research) and to facilitate online completion of the questionnaire. The final questionnaire took approximately 5–10 minutes to complete.

In February 2006, a link to an online questionnaire was emailed to 1,100 IN-CAM members and 165 members of the CAM Education and Research Network of Alberta (CAMera). Membership in these networks is not a formal paid membership but consists of signing up in a membership database in return for which 'members' receive the networks' newsletters and newsflashes (emails). IN-CAM and CAMera members include CAM researchers, practitioners, educators, policy makers and students. One separate email describing the objectives and process of this survey was sent to all members of each network within the span of one week. There is some overlap in membership between the networks, so it is possible that some individuals received the notification more than once.

The majority of survey questions were open-ended (Table 1). Other questions addressed participants' role (e.g., practitioner, researcher), involvement in CAM research, use of outcome measures and sources of information on outcome measures used. Validity, reliability, responsiveness, relevance, cost and patient and administrator burden are criteria commonly applied when selecting outcome measures for use in clinical research [15]. We asked respondents to identify assessment criteria they apply in addition to this list.

**Table 1:** Sample Questions Asked on Electronic Questionnaire

	**Question**
1.	Many outcome domains have been identified as important for CAM research that fall into the broad categories of biological, physical (e.g., function, pain), mental (e.g., depression, anxiety), social (e.g., role function, social support), emotional (e.g., hope) and spiritual outcomes domains.
	Are there any OTHER outcomes of CAM interventions you feel are important and should be included in CAM research (please describe)?
2.	What CAM outcome measures would you like to use but have not been able to find (please describe)?
3.	Common assessment criteria that are used when selecting outcome assessment tools include validity, reliability, responsiveness, relevance, burden and cost.
	Are there any OTHER assessment criteria that you use to assess measurement tools (please describe)?
4.	What do you perceive to be barriers to finding outcome measures for CAM research (please describe)?

Analysis of responses to the open-ended questions was conducted using qualitative content analysis: responses to each open-ended question were first read as a complete list and then common responses were sorted into a set of data-derived categories. The goal of the analysis was, for each question, to summarize the range of responses and to suggest the importance of some categories over others based on frequency of mention.

Data from a question soliciting respondents' perceptions of important CAM outcome domains (Table 1, Question 1) was used to develop a preliminary framework of outcome domains important for CAM research. The framework emerging from the data was very basic and relationships between the domains were not specified. Subsequently, this framework was refined through a consensus building meeting attended by all authors and through consultation with experts in the field of CAM and outcome assessment.

## Results

A total of 164 responses were received. The largest number of respondents identified themselves as practitioners (32.3%) followed by researchers (28%) and educators (12.8%). Approximately half (48.2%) indicated that they are actively involved in CAM research, of which 78% use outcome measures in their research (Table 2).

**Table 2:** Characteristics of Survey Respondents

	n	%
Primary Role*:		
Practitioner	53	32.3
Researcher or Research Staff	46	28.0
Educator	21	12.8
Student	16	9.8
Administrator	8	4.9
Other	16	9.8
Actively involved in CAM research*:		
Yes	79	48.2
No	81	49.4
Currently use outcome measures in research:		
Yes	62	78.5
No	17	21.5

* 4 respondents did not answer this question

### Resources for outcome measures

Respondents indicated that they most commonly refer to colleagues as a resource for outcome measures (34.4%), followed by libraries (27.4%), and the Internet (22.3%). PubMed was identified most frequently as an Internet site to help identify outcome measures. Google, the Cumulative Index to Nursing and Allied Health Literature and the National Center for Complementary and Alternative Medicine (United States) website were also mentioned, but less frequently.

### Use of outcome measures

The 62 respondents who reported using outcome measures in their CAM research identified 92 different specific outcomes, of which 63 were identified by name. The remaining identified outcome measures were biological measures such as heart rate, blood pressure and range of motion, or general concepts, such as satisfaction, control and attitudes but with no specific tool name mentioned. The most commonly identified tools were: Short Form 36 Health Status Survey (SF-36: n = 9), Profile of Mood States (POMS: n = 7), Arizona Integrative Outcomes Scale (AIOS: n = 5), pain visual analogue scales (pain VAS: n = 4) and Measure Your Medical Outcome Profile (MYMOP: n = 4). Some of these outcome measures are also frequently used in conventional medical research (e.g., SF-36, pain VAS); however, others were specifically developed for and validated in CAM patient populations (e.g., AIOS, MYMOP).

### Assessment criteria for outcome measures

Many assessment criteria were identified in addition to the provided list, including: accessibility (e.g., ease of locating, copyright issues), ease of use and scoring, extent of use, readability, overall look and word choice.

### Barriers to finding outcome measures

Respondents identified many barriers that they face when trying to find outcome measures for their CAM research. The most commonly identified barrier was that appropriate and relevant outcome measures do not exist (n = 16). Respondents stressed that outcomes in CAM research deviate somewhat from those in conventional medical research and that measurement tools have not yet been developed to address the unique process, context and outcomes of CAM interventions. Closely related to this barrier was the belief that many of the concepts important to CAM research have not yet been explicitly defined (n = 8), for example personal transformation [16] or unstuckness [17]. Other identified barriers included: a lack of psychometric data on existing tools (n = 11); lack of a common resource of outcome measures (n = 7); not knowing where to look (n = 5); poor documentation regarding outcome measures used in published research, making comparative studies difficult (n = 4); copyright issues (n = 2); and prohibitive cost (n = 2).

### Important domains of outcome measures

Participants responded in many different ways to the question regarding important outcome domains for CAM research. Many identified that it is important to look at different perspectives of outcome measures such as the patient, provider, family and community perspective. Others identified aspects of interventions other than outcomes, such as process, context and meaning. An additional group provided examples of outcome measures that they might have found difficult to group in domains, such as serenity, integration, body image, ability to relax, awareness and holism.

### A framework of outcome domains important in CAM research

Based on the questionnaire responses, a consensus building meeting of the authors and feedback from experts in CAM and outcomes assessment, we have developed a preliminary framework of outcome domains important in CAM research (Table 3). The framework has nine main categories. Outcomes can address physical, psychological, social and spiritual aspects of the intervention. Holistic outcomes encompass each of the physical, psychological, social and spiritual domains. Quality of life, encompasses any two of the physical, psychological, social and spiritual domains. Individualized measures assess unique patient-centred outcomes for each research participant. Examples of such measures are the MYMOP [18] and Goal Attainment Scaling [19]. While individualized measures may be classified into any or all of the remaining domains, depending on the nature of a research participant's identified health concerns and personal treatment goals, they are unique because of their individualized nature and were therefore placed in a separate outcome domain. The context of healing and the process of healing are not outcomes *per se *but were identified as being of such importance to CAM research that, for completeness, they are included in our framework.

**Table 3:** Preliminary Framework of Outcome Domains Important for CAM Research

**Holistic wellness/well-being (encompassing each of physical, psychological, social, spiritual)**			
**Physical**	**Psychological**	**Social**	**Spiritual**

Biological markers	Absorption	Adjustment	Awareness
Disability	Anger	Advocacy	Balance
Energy	Anxiety	Economic	Enablement
Fatigue	Attitudes and Beliefs	Health care utilization	Energy
Function/Activities of Daily Living	Awareness	Cost-effectiveness	Harmony
Pain	Coping	Relationships	Hope
Pathology	Depression	Role function in daily life and work	Peace
Sleep	Empathy	Social support	Relaxation
Symptom management	Enablement	Socioeconomic	Spirituality
	Energy	Social Strain	Transformation
	Patient expectations	Religiosity	
	General		
	Hope		
	Introversion		
	Locus of control		
	Mood		
	Openness to experience		
	Optimism/Pessimism		
	Patient knowledge		
	Patient motivation		
	Patient perceived self-efficacy		
	Patient perceptions of care		
	Patient perceptions of risk		
	Patient preference for control		
	Patient Satisfaction		
	Readiness		
	Relaxation		
	Resilience		
	Self-Esteem		
	Sense of Coherence		
	Stress		
	Trust		

**Quality of Life (assessing at least two of physical, psychological, social, spiritual)**			

Global			
Multidimensional			

**Individualized**			

e.g. Measure Your Medical Outcome Profile, Measure Your Concerns and Well-being, Goal Attainment Scaling			

**Context of healing**			

Healing environment			
Negotiations between patients and practitioners			
Patient-centeredness of healing			
Patient expectations			
Patient-practitioner relationship			
Practitioner attitudes towards integration			
Practitioner attributes			
Practitioner expectations			
Practitioner experience			
Attunement			
Therapeutic Intent			

**Process of healing**			

Adjustment			
Engagement with intervention experience			
Personal growth			
Transformation			
Unstuckness			

Table 3 identifies each main category heading and lists sub-concepts to describe specific outcomes in that domain. For example, awareness, balance and enablement are fundamental concepts within the spiritual domain. Awareness, however, may also be conceptualized as a psychological outcome and for this reason is listed under both the spiritual and psychological domains. Although the nine categories are not mutually exclusive, our aim was for as much exclusivity as possible. Many relevant concepts, by their nature, address more than one clinically relevant outcome domain and therefore exclusivity appears impossible.

**Figure 1:**
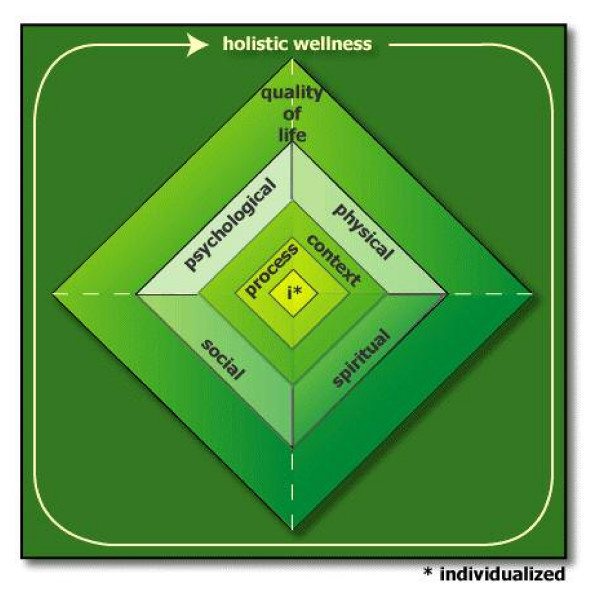
Framework of Outcome Domains.

Presenting our framework of outcome domains as a table does not adequately portray the linkages between domains, nor the underlying global concepts of whole systems and healing; however, we are somewhat constrained by a two-dimensional manuscript. A diagram is somewhat more useful, as it can highlight these linkages, although it cannot show the amount of detail that can be listed in a table. A complementary figure to Table 3 is presented in Figure 1. In the figure, outcomes that are more specific are indicated towards the centre, and outcomes that are more general towards the outside. Quality of life has dashed lines at the four corners to indicate that it may comprise some or all of the four categories it contains. The choice of colour also intends to infer meaning: green commonly symbolizes holism; quality of life is a gradient of the four colours representing the four categories; process and context fade toward the four categories; and bright yellow represents individualized outcomes to indicate difference from the rest.

## Discussion

Our preliminary framework provides a foundation for the development of a large, comprehensive collection of CAM outcomes. The framework fits very well in a whole systems perspective, which requires an expanded set of outcome measures. Several authors have previously conceptualized the implications of a whole systems approach to the range of potential patient-related outcomes [14,20,21], although it was not their intent to develop a framework of CAM outcomes. All identified the importance of the process and context of an intervention, in particular the patient-provider relationship. Each also addressed the impact of the philosophy and practice of health and healing on the therapeutic relationship, for example assisting patients in self-healing and providing individualizing treatments. Our framework builds on this work by highlighting how all relevant outcome domains, including issues of process and context, are linked and encompassed by a consistent philosophical approach to health and healing. Although measures do not exist for every outcome domain in the framework, the framework can be used to identify "gaps" and to direct future outcome measure development.

Our proposed framework may also be used when making decisions regarding which outcome measures to use to ensure all appropriate domains are considered. The framework and the data from this survey will be used to develop an online database of outcome measures relevant to CAM research. The database will help address some of the identified barriers to finding outcome measures – for example, the lack of a common resource. Further, appropriate marketing and hosting the database on IN-CAM's web site [22] may address the barrier of not knowing where to look.

Undoubtedly, the question will be posed whether there is a need to make such a clear distinction between conventional and CAM outcomes and whether we are not, in fact, adding to the polarization between conventional medicine and CAM. We do not believe this is the case, and would encourage a similarly broad perspective in conventional medical research. It is easy to see how conventional interventions can also be multidimensional, placing value on the patient-provider relationship and acknowledging non-specific aspects. For example, the assessment of treatment interventions in cancer care often falls short in addressing the complexity of cancer treatment outcomes. Whether or not CAM is included as part of treatment, cancer treatment consists of many component parts that are difficult to tease apart, such as support from friends and family, the degree to which information needs are met and a strong patient-provider relationship. Each component plays an essential role in patient healing and when considered as a whole system of care, the whole is much greater than the sum of the component parts. All effectiveness research, whether CAM or conventional, should be designed to capture all factors relevant to the intervention and all relevant outcomes. Other common examples of conventional interventions that may be regarded as whole systems are palliative care, addiction treatment and chronic pain management.

Our outcomes are particularly relevant to the concept of Integrative Medicine, which ideally covers the entire spectrum of 'good medicine' [23,24]. Currently, many CAM and integrative medicine practitioners do not use outcome measures to assess their interventions and, further, many CAM/IM researchers limit themselves to biomedical and quality of life outcomes. Therefore, it is important that comprehensive information is available to encourage the use of psychometrically sound and relevant outcome measures in CAM/IM research and practice. We hope that the availability of an outcomes database will assist CAM/IM researchers in refining and clarifying their research questions, which too often are broad and unspecific. The framework highlights the fact that there are many potentially relevant outcomes of CAM/IM interventions. Ideally, research should encompass all relevant domains; but, the decision of which outcome measures to use must balance the choice between an all-encompassing set of outcome measures of high psychometric quality and patient burden. Further work on identifying a core group of outcomes for CAM research is clearly needed.

Some limitations to our study must be acknowledged, specifically with respect to our sampling method and response rate. First, we conducted an online survey of a motivated group of individuals interested in CAM research. Each member of our sampling frame has access to the Internet and had taken the time to sign up as a member of IN-CAM, CAMera or both. This group is therefore likely not representative of all individuals in Canada with an interest in CAM research. Further, our response rate was relatively low when standard survey methodology is considered; however it is the highest response rate received for an online survey conducted by either of the research networks. Since membership is free of charge and there are no requirements for network membership, it is to be expected that our survey was relevant to only some of the 'members'. Our respondents are clearly, then, a highly motivated group of individuals interested in CAM outcomes and may not be representative of CAM researchers and practitioners as a whole. We believe, however, that 164 responses, plus the length of many written responses, suggest the relevance of our project to those involved or interested in CAM research. The magnitude of the response also suggests that the area of CAM outcomes is in need of development and clarification.

## Conclusion

A publicly available resource for accessing outcome measures that are relevant for CAM research is needed. We propose a framework for considering outcomes for clinical research into CAM from a whole systems perspective. We invite comments from readers regarding our framework and the database development. If you would like to be included on a list to remain updated about the progress of the database development, send the corresponding author an email.

## Competing interests

The author(s) declare that they have no competing interests.

## Authors' contributions

All authors made substantial contributions to the conception and design, acquisition, analysis and interpretation of data. LV prepared the first draft and all other authors were involved in drafting the manuscript and revising it critically for important intellectual content. All authors read and approved the final version of the manuscript.

## Pre-publication history

The pre-publication history for this paper can be accessed here:


